# On the Emergence and Awareness of Auditory Objects

**DOI:** 10.1371/journal.pbio.0060155

**Published:** 2008-06-24

**Authors:** Shihab Shamma

## Abstract

How do humans successfully navigate the sounds of music and the voice of a friend in the midst of a noisy cocktail party? Two recent articles in*PLoS Biology* provide psychoacoustic and neuronal clues about where to search for the answers.

Anyone who has walked into a crowded reverberant nightclub, with a hubbub of multiple conversations amidst blaring music, will recall the initial impression of the sound as loud and undifferentiated noise. In short order, however, different sound streams begin to emerge as one attends to individual speakers, listens to the melody from the band, or even hears one instrument in it. Humans perform this remarkable feat effortlessly. Our extraordinary abilities to extract signal from noise have evolved in natural environments that are often extremely auditorily cluttered. Many animals have developed abilities to navigate their complex auditory scenes in order to mate, locate prey, feed their young, and avoid predators. It is likely that these abilities are mediated by similar mechanisms that have evolved in many animals and include a mix of “bottom-up” automatic processes with complex “top-down” behaviors involving attention, expectation, learning, and memory (as illustrated schematically in Figure 1). However, little is known about the underlying computational details, or the manner in which these diverse processes interact to give rise to this auditory ability. And it is therefore no coincidence that we still lack engineering systems that can recognize speech robustly in realistic environments, or reliably transcribe polyphonic music.

**Figure 1:**
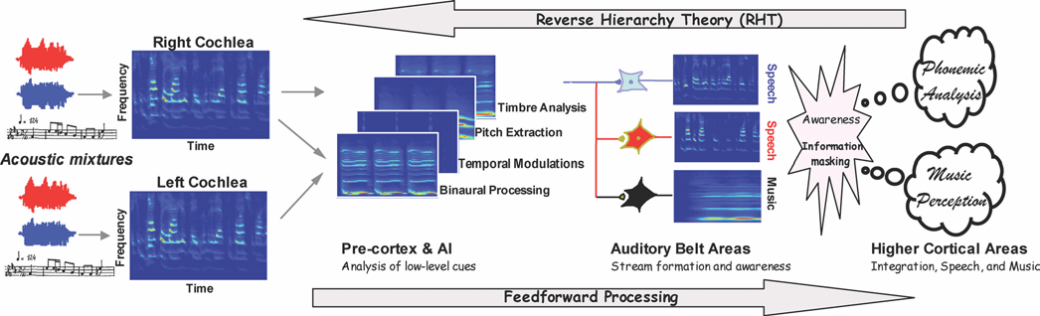
Bottom-Up Flow and Top-Down Control of Information in the Auditory System Schematic of the bottom-up feed-forward flow of auditory analysis and the top-down cognitive influences (RHT) that give rise to auditory perception and awareness. From left to right, natural acoustic scenes usually contain mixtures of multiple speakers (red and blue signals) and music. Low-level cues embedded in the cochlear spectrograms from the right and left ears are analyzed and combined in several precortical and primary auditory cortical (A1) stages. Neural correlates of consciously perceived streams of speech and music would emerge in the auditory belt areas beyond A1. In complex realistic scenes, ambiguous (“informationally masked”) speech and musical streams are resolved through top-down influences described by the RHT.

The neural underpinnings of attention, feature selection, object binding, and other perceptual and cognitive phenomena have been the focus of research in the visual system. However, addressing these phenomena in the context of auditory perception promotes unique perspectives that stem from the temporal nature of sensory signals in animal communication, human speech, and music. For instance, in auditory perception research, “auditory objects” are almost never thought of as static images, but instead as “streams” that build up over time to form a particular speaker's voice in a crowd or the music of an orchestra's string section. The rules and interactions between the stream percepts and the low-level cues that group the elements of a stream and distinguish it from its counterparts (e.g., pitch, timbre, and binaural cues) have been delineated over the years under the umbrella of auditory scene analysis (see Glossary) [1]. Equally important is the role of attention in promoting stream formation [2], and also the opposing processes that hinder the emergence of a target stream, known collectively as informational masking [3–5]. Here, distractors do not interfere through classic “energetic masking” of low-level cues, as would happen with broadband noise competing with a signal filtered through the same cochlear channel. Instead, noises act as potential distractor streams that confuse the listener and compete for his or her attention at a fairly high level of representation (e.g., different words that are semantically similar).

**Glossary**

**Auditory scene analysis:** A term originally coined by Bregman [1] that refers to the suite of auditory processes invoked to explain how humans and animals organize the perception of complex acoustic environments into different sources.

**Energetic and informational masking: Energetic masking** is the phenomenon by which one sound hinders the perception of another because they compete for the same neural channel. In the auditory system, the best-known example of energetic masking is among sounds that share the same cochlear filters.

**Informational masking** is interference among sounds that do not share the same cochlear filters. It is also associated with the increased difficulty of attending to one stream among many in an acoustically cluttered scene.

**Ideal observer detection:** An ideal observer model is the algorithm that yields optimal performance, given all the stimulus cues and the constraints of the task.

**Object binding:** This term refers to the process of integrating all features (e.g., color, shape) of an object to form a single, unified percept of that object.

**Streams:** Streams serve the role of “objects” in the auditory perception of acoustic scenes. Like visual objects, streams differentiate and coexist based on a multitude of attributes that give each stream its distinctive perceptual identity. These attributes include timbre, pitch, location, and loudness.

Two new articles in *PLoS Biology* [6,7] explore and expand on these themes and lead the auditory research community into uncharted territory, presenting creative opinions, provocative psychoacoustic results, and exciting neuronal recordings. These studies will likely have significant impact on, and implications for, the theoretical and experimental research into the biological mechanisms underlying scene analysis and informational masking. The two papers complement each other well in techniques (psychoacoustics versus behavioral magnetoencephalography (MEG) recordings), scope of their exploration (precortical and cortical phonemic levels versus the primary auditory cortex [A1] and its immediate belt), and their fundamental findings (the conditions that facilitate object formation versus the neural correlate of perceptual awareness of object formation).

The paper by Nahum and colleagues [6] provides the first thorough test of the reverse hierarchy theory (RHT) in auditory perception. This theory has been influential in guiding research on the perception of natural visual scenes and the way in which low-level cues are utilized in parsing complex scenes [8,9]. It postulates that a parsing decision is first based on the highest available level of visual representation (e.g., objects). If the discrimination task is poor at that level (because of the reduction in sensory resolution that usually accompanies the generalization process), it proceeds down the representational hierarchy to benefit from more detailed, lower-level cues that participate in generating the percept. If the high “objects” and their “low-levels cues” are congruent, the feed-forward process is rapid, and use of all available salient cues is effective and comprehensive (as would be the case for the “ideal observer”). But if the two are incongruent (e.g., as in highly similar, overlapping (all male) voices and faces), the RHT postulates that the backward interrogation and integration of low-level cues requires special conditions and can be easily disrupted by diverting attention to other representational levels, or even to competing cues. Such disruption leads to failure to make use of all available sensory cues (no longer an “ideal observer”), as in fact has long been experimentally demonstrated [4]. Thus, the Nahum et al. article [6] provides a model of how higher representations interact with low-level cues in stream formation, and explains why “ideal observer” and “limited attentional resource” models [10] fail in certain circumstances.

But where do low-level cues end and high-level cues begin? In the visual system, the sensory signal is passed through the retinal circuitry to the lateral geniculate nucleus and thence to the primary visual cortex, where much of what is termed low-level processing begins to occur. In the auditory system, by contrast, sensory signals pass through multiple stages and transformations on the way from the cochlea to A1. Although it is well accepted that correlates of energetic masking can be found in the auditory periphery [11], the locus (or loci) of informational masking has remained a mystery. The second paper, by Gutschalk and colleagues [7], takes this theory a step further and describes behavioral experiments coupled with MEG recordings that demonstrate, for the first time, a neural correlate of the perceptual awareness of a target sound, localizing it beyond the primary core auditory cortex (in the secondary auditory belt). The behavioral task employs a stimulus used in auditory research on informational masking [5,12]. It is the auditory equivalent of a cloud of randomly dispersed and desynchronized arrhythmic flashes (tones) surrounding a regularly flashing and spatially slightly-segregated target (tone) sequence that, consequently, may “pop out” of the complex scene. In the experiment, the regularly repeating target tones may be present or absent on any given trial, and the subject's task is to indicate as soon as he/she detects their presence. By contrasting neural responses to target tones that were detected by the listener from responses to undetected target tones, the authors identified a prominent variable long-latency response (50–250 ms) to detected (but not undetected) targets; they called this response the “awareness related negativity”. This finding demonstrates a correlation between the conscious detection of a sound stream and neural activity beyond the primary (or core) auditory cortex.

In contrast, both detected and undetected targets produced equally robust auditory middle-latency steady-state responses, known to originate in the primary auditory cortex [13]. This key result establishes a clear correlation between a perceptually parsed auditory target (a stream) and a neural correlate that reliably appears only when this stream is consciously perceived. It also indicates that while the primary auditory cortex responds to the whole (undifferentiated) stimulus regardless of whether it is detected or not by the listener, the secondary auditory cortex represents awareness of the target, i.e., a change in the auditory scene as the target becomes the object of conscious perception and is differentiated from the stimulus.

These two exciting studies will likely inspire new directions for research on the neural mechanisms of auditory scene analysis and perception of speech and music. Such an endeavor needs theoretical formulations (exemplified by the RHT) that transcend the simple ideal observer models that served us well in the detection and discrimination of simple stimuli. Future theories will also have to be more closely intertwined with biological facts (neuronal mechanisms and architectures) that are at present simply lacking in the auditory system, and that will only emerge from animal experimentation guided and inspired by MEG, functional MRI, and psychoacoustics with humans [14]. But for animal experiments to unravel the mysteries of complex sound perception, it is essential that they encompass the study of auditory attention, memory, and plasticity, preferably while the animals are engaged in appropriately complex behaviors—just as we are while participating in cocktail party chatter. Humans maintaining a conversation while navigating the acoustic clutter of a cocktail party rely on such processes as attention to a host of auditory and visual cues and retrieval of memorized linguistic and contextual information, as well as rapid adaptation of their receptive fields to the peculiarities of the environment and the accent of the speaker.

Such approaches are common in experiments with animal vision, but are still rare in auditory research. One reason for this dearth is the absence of widely accepted orderly neural representations of higher auditory features (analogous to orientation, motion, and color selectivity in vision) that can serve as assays for the neural correlates of attention and learning in the auditory cortex. There is also a lack of clear understanding of the physiology and functional organization of secondary auditory fields beyond the primary auditory cortex (analogous to the V2, V4, MT, MST, FEF, and IT areas in vision [15]). And finally, there is still much to be discovered about the anatomical connections within the auditory fields, and between the auditory fields and structures serving higher levels of representation, such as the prefrontal cortex.

However, the finding reported by Gutschalk et al. [7], that neural correlates of auditory awareness are likely situated beyond A1, is sure to provide an impetus for a more concerted exploration of the secondary auditory fields and higher areas using natural stimuli, complex behaviors, and a broader range of complex percepts. For example, bistable auditory percepts (analogous to binocular rivalry [16]), which are common when listening to competing streams, share similar dynamics and statistical properties with their visual counterparts, and hence may provide new viewpoints and insights into the underlying attentional mechanisms and their dynamics [17]. Another likely implication of the finding on auditory awareness is its relation to the widely recorded mismatched negativity potential in humans and animals, which signals a “deviant” in a sequence of simple or complex patterns of sound, seemingly even when subjects are unaware of its occurrence [18].

Speech and music are complex acoustic signals, but above all they are unique in being produced by the human mind. As such, understanding their auditory processing and representations could potentially offer rich glimpses into the workings of the mind. These two papers serve as significant mileposts along this long and exciting, but arduous journey.

## Abbreviations

MEG: magnetoencephalography
RHT: reverse hierarchy theory
